# microRNA-22 can regulate expression of the long non-coding RNA MEG3 in acute myeloid leukemia

**DOI:** 10.18632/oncotarget.18059

**Published:** 2017-05-22

**Authors:** Hongxia Yao, Pei Sun, Mengling Duan, Lie Lin, Yanping Pan, Congming Wu, Xiangjun Fu, Hua Wang, Li Guo, Tianbo Jin, Yipeng Ding

**Affiliations:** ^1^ Department of Hematology, Hainan General Hospital, Haikou, Hainan 570311, P.R. China; ^2^ Department of Hematology, Hunan Yiyang Central Hospital, Yiyang, Hunan 413000, P.R. China; ^3^ Key Laboratory of Resource Biology and Biotechnology in Western China (Northwest University), Ministry of Education, Xi’an, Shaanxi 710069, P.R. China; ^4^ Department of Emergency, Hainan General Hospital, Haikou, Hainan 570311, P.R. China

**Keywords:** acute myeloid leukemia(AML), MEG3, long non-coding RNA, TET2, miR-22-3p/5p

## Abstract

**Aim:**

Acute myeloid leukemia (AML) is the most common blood tumor with poor prognosis. At present, the research found that the pathogenesis of AML is related to many factors, such as recurrent somatic mutations and gene expression and epigenetic changes, however, the molecular mechanism of AML is still unclear. Long non-coding RNA MEG3 is a newly found tumor suppressor and plays a very important role in the regulation of a variety of tumor formation and progression. Studies found that the MEG3 expression was significantly decreased in AML. However, to date, it is not clear the cause of its abnormal expression. Therefore, the molecular mechanism of AML is urgently needed to study the molecular mechanism of AML.

**Methods:**

The different expression level of MEG3, TET2, miR-22-3p, miR-22-5p in AML was detected by real-time quantification PCR. MEG3, TET2, miR-22-3p, miR-22-3p expression cell pools in K562 cells was used to interfering and TET2, MEG3 TET2, relations with miR-22-3p, miR-22-5p. The effect of AML cell on proliferation was evaluated by TET2 lower expression.

**Results:**

1. The lower expression of MEG3 and TET2 in AML cell lines was detected by RT-qPCR. 2. The stable MEG3, TET2 overexpression cell pools in K562 cells was successful established. 3. After transfection, MTT assay revealed that cell growth was significantly increased in AML cell lines transfected with TET2 compared with controls.

**Conclusions:**

Our findings suggested that MEG3 is significantly down regulated in AML cell lines.

## INTRODUCTION

Acute myeloid leukemia (AML) is the most common blood tumor. In recent years, with the rapid development of genetics, molecular biology and sequencing technology, more in-depth understanding of the pathogenesis has been made. Improvements in chemotherapy regimens and supportive therapies, as well as the widespread use of novel drug delivery and hematopoietic stem cell transplantation have significantly improved patient outcomes and outcomes. However, more than young patients and older patients died AML [1]. Moreover, with the industrial development and other factors, the incidence of AML showed an increasing trend year by year [2]. The study found that mutations in AML lead to the abnormal regulation of several signal transduction pathways, the potential target is very fragmented [3], therefore, in-depth study of the molecular mechanism for the urgent need of AML, in order to obtain more biomarkers and specific segments or a new target for the treatment.

Long-chain non-coding RNAs(lncRNAs) are non-coding RNAs with a transcription length of more than 200nt, which can regulate chromatin remodeling, histone modification and DNA methylation, and have a profound impact on the development of tumors and other diseases [4]. There is evidence that the expression levels of some lncRNAs are significantly altered in specific malignancies, and the abnormal expression of such lncRNAs can be a diagnostic marker and potential drug target for a particular tumor [5–7]

lncRNA MEG3, a newly discovered lncRNA with tumor suppressor function, plays an important role in the formation and progression of many tumors. The study found that MEG3 in acute myeloid leukemia (AML) in the expression significantly decreased, but its influence on the biological behavior of AML tumors is still unclear. In various tumor tissues, the expression of MEG3 decreased significantly in brain, bladder, breast, cervical, colon, bone marrow, liver, lung and prostate cancer cells in the expression decreased obviously [8–10]. Since MEG3 can promote the binding of tumor suppressor gene P53 to target, the down-regulation of expression may promote the proliferation of tumor cells [8, 11, 12]. In nearly half of the AML, the content of MEG3 decreased significantly [13]. But until now, whether MEG3 activity can affect the growth of AML cells and whether the mechanism can affect the growth of AML cells is not clear.

TET2 gene mutations have been found in a variety of bone marrow malignancies, including acute myeloid leukemia, chronic myelomonocyticleukemia, myelodysplastic syndrome, polycythemiavera, primary myelofibrosis, idiopathic thrombocytosis, mastocytosis et al. [14, 15]. TET protein is a member of the DNA family can catalyze 5- hydroxylase, methyl cytosine demethylation, has very good characteristics of epigenetic modifications, play an important role in regulating gene expression and maintaining cell surface marks [16, 17]. Methylation of DNA is an important force to promote tumor formation and malignant progression, 5hmC is in the TET2 enzyme catalyzed by 5mC oxidation, 5hmC is a metabolic product of 5mC, which will lead to a decline in 5mC, and demethylation. Thus, reduced TET2 activity leads to changes in DNA methylation patterns (such as promoter hypermethylation)[18]. TET2 inactivation is also an important reason for promoting the occurrence and development of AML. Its inactivation may lead to the demethylation process of DNA damaged, so that the tumor DNA at least in some areas of hypermethylation, and ultimately promote tumor development [19].

The study found that miR- 22 can regulate the expression of TET2 negative, thus reducing the expression of 5 - hydroxy methyl cytosine and enhance methylation of multiple genes.

So, in our study we will to study TET2 inactivation and MEG3 methylation of correlation. To explored the causes of TET2 deactivation to clarify cause MEG3 molecular mechanism of deactivation.

## RESULTS

### ExpressionofTET2, MEG3, miR-22-3p, miR-22-5p

**Figure 1:**
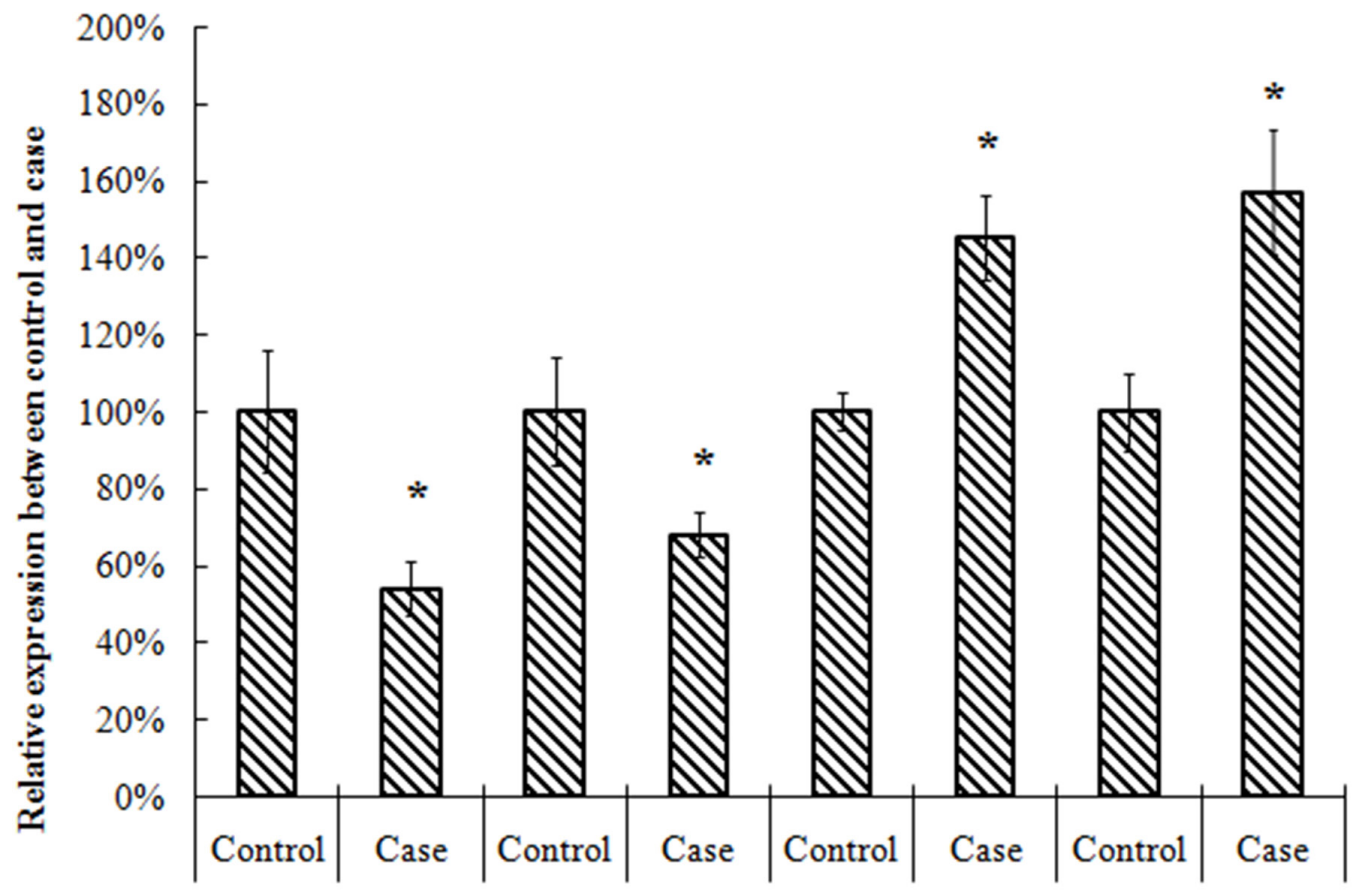
Expression of TET2, MEG3, miR-22-3p, miR-22-5p in AML and control

To explore the role of TET2, MEG3, miR-22-3p, miR-22-5p in AML, we analyzed 20 AML and normal counterparts for expression using qRT-PCR. In Figure 1, we found TET2 and MEG3 was signficantly decreased in tumor samples, however, miR-22-3p, miR-22-5p was signficantly increased in tumor samples.

### Expression of TET2 gene and MEG3 gene in K562 cells transfected with TET2-siRNA

**Figure 2:**
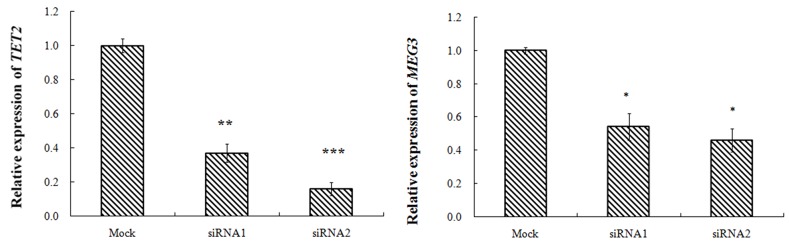
K562 cell transfection TET2-siRNA, MEG3 and TET2 gene expression

**Figure 3:**
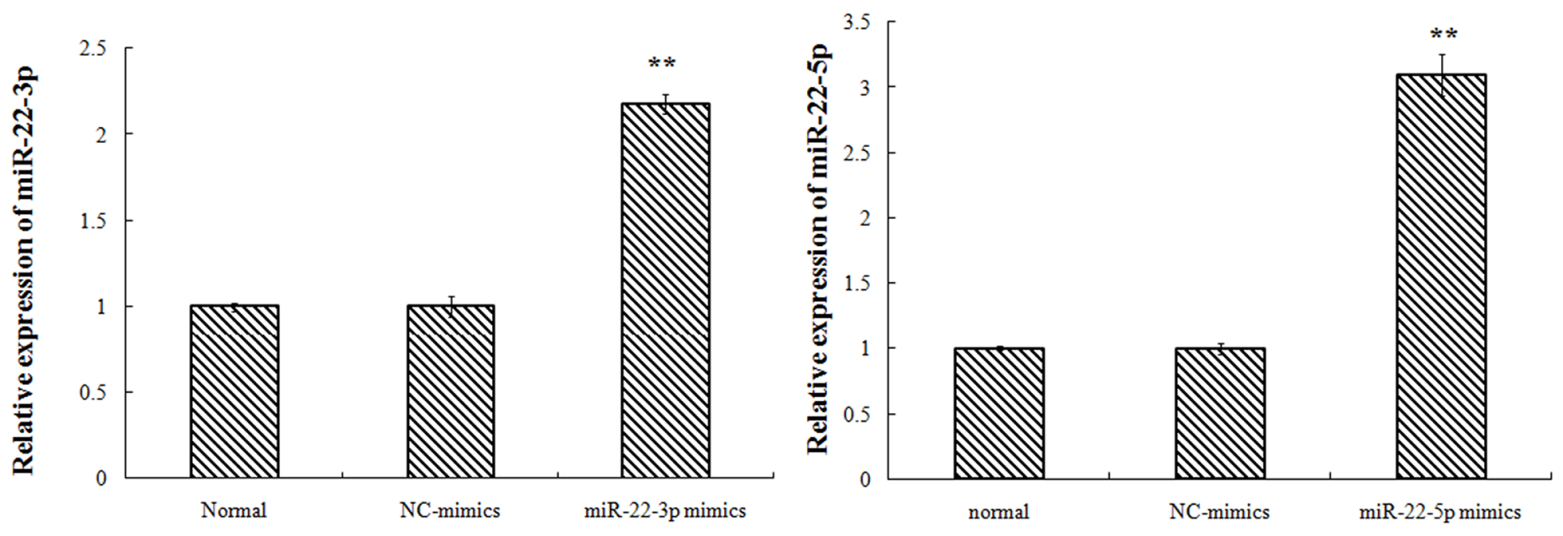
K562 cell transfection miR-22-3p/5p, miR-22-3p/5p expression (the nucleic acid level)

**Figure 4:**
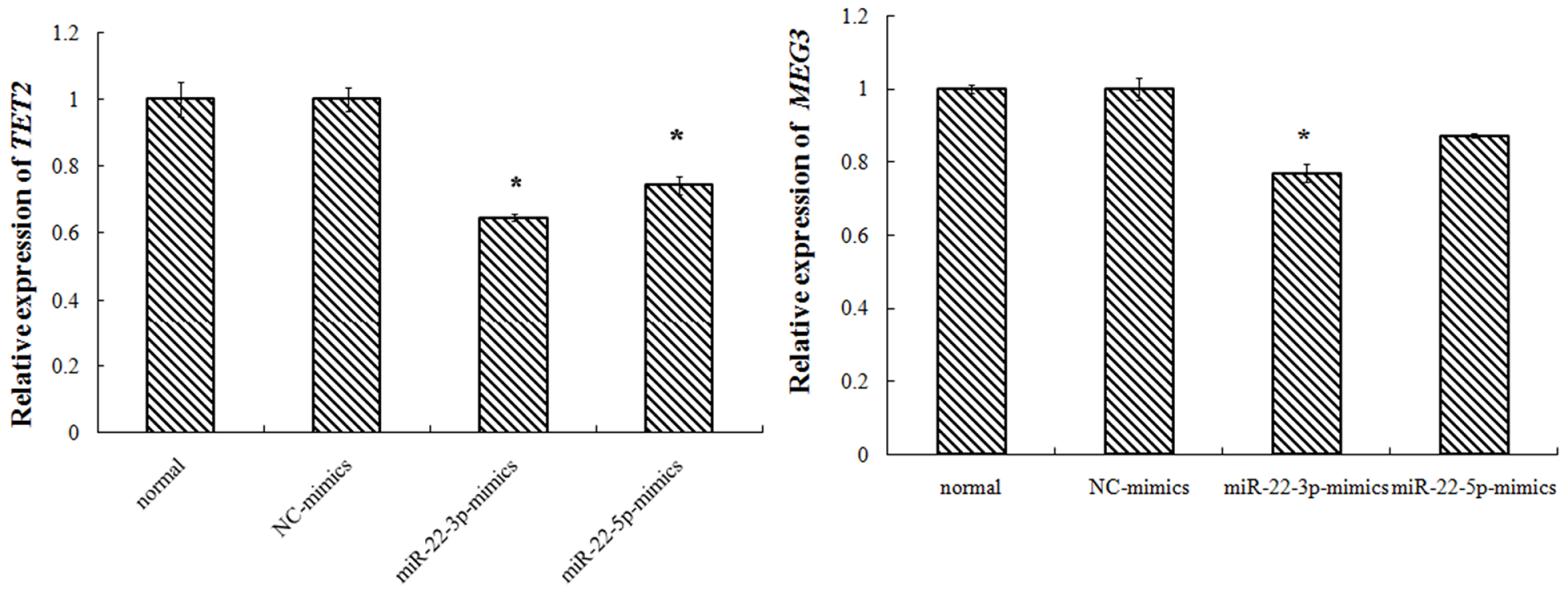
K562 cell transfection miR-22-3p/5p, miR-22-3p/5p expression

Transfected with TET2-siRNA, compared with K562-NC group, the TET2 expression in siRNA1, siRNA2 group was 37%, 16% respectively with difference of statistical significance. Compared with K562-NC group, the MEG3expression in siRNA1, siRNA2 group was 54%, 46% respectively with difference of statistical significance. Transfected with miR-22-3p/5p mimics, and the results showed that the expression of miR-22-3p/5p mimics was obvious. Transfected with miR-22-3p/5p mimics, compared with NC-mimics group, the TET2 expression in the miR-22-3p-mimics, the miR-22-5p-mimics group was 65%, 97% respectively with difference of statistical significance. Compared with NC-mimics group, the MEG3 expression in miR-22-3p-mimics, the miR-22-5p-mimics group was 77%, 87% respectively with difference of statistical significance (Figures 2–4).

### Detection of cell reproductive capacity

**Figure 5:**
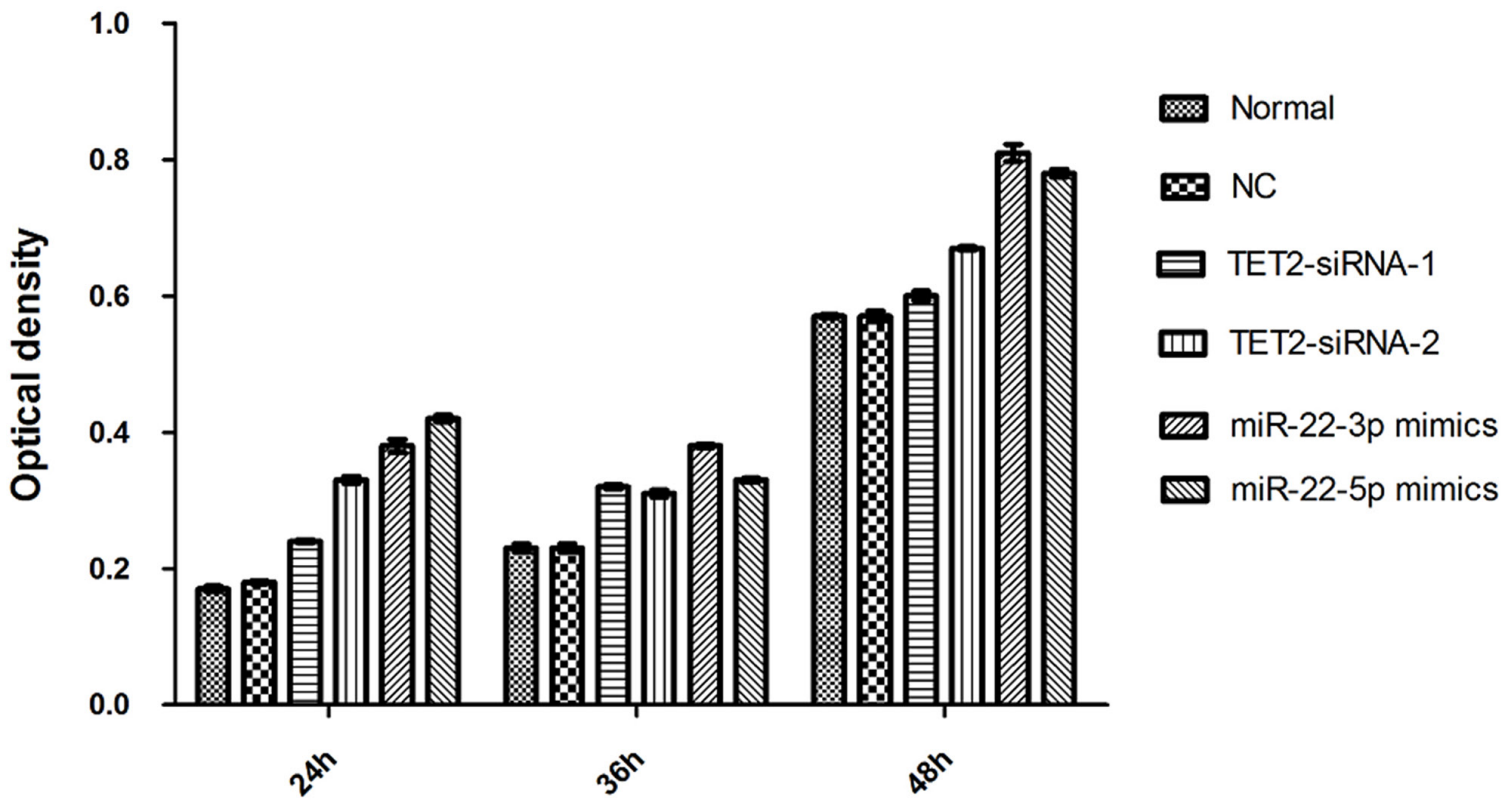
Detection of cell reproductive capacity

The change of proliferation capacity of stable cell strains, TET2, miRNA-22-3p/5p siRNA infection was detected by CCK-8 method. The results showed that the inhibition ratios of cellular proliferation in TET2-siRNA1 group, TET2-siRNA2 group, miRNA-22-3p mimics group andmiRNA-22-5p mimics group were increased. Above results showed that TET2 and MEG3 lower expression can promote the AML proliferation (Figure 5).

## DISSCUSION

Some studies have shown that lncRNAs can regulate multiple signaling pathways in the process of development, and the disorder of lncRNAs regulation may result in the development of tumors by affecting the epigenetic information. Although lncRNAs has an important effect on the occurrence of tumor, its mechanism is still unclear. MEG3 is one of the lncRNAs [20]. It was discovered in 2000 by Miyoshi et al., located on chromosome 14q32.3, the mature MEG3 RNA length is about 1600nt [20]. Zhang et al. [11] used in situ hybridization to detect various tissues of human body, and found that MEG3 was expressed in normal tissues such as brain, pituitary, placenta, adrenal gland, pancreas and ovary. However, MEG3 expression missing was found in a variety of tumor cell lines, including brain, bladder, bone marrow, breast, cervix, colon, liver, lung and prostate [8, 11, 12, 21, 22]. Overexpression of MEG3 gene can inhibit the proliferation of tumor cell lines and act as a tumor suppressor gene. Expressing MEG3 gene has been reported is caused by multiple mechanisms lack in human tumors and tumor cell lines, including the regulation of transcriptional activity mediated by P53 gene [23], promoter region CpG island methylation [24] and demethylation effect [22]. In several cancers, the MEG3 promoter was confirmed to have aberrant methylation in the CpG Island [23]. Hypermethylation in 43 patients with myelodysplastic syndrome and 42 cases of acute myeloid leukemia patients were 34.9% and 47.6% of the patients with MEG3 promoter, further found that hypermethylation of MEG3 promoter in patients with poor prognosis [13]. These studies suggested that the inactivation of MEG3 gene may be due to the hypermethylation of its promoter. In nearly half of the AML, the content of MEG3 decreased significantly [8]. However, it is not clear whether the decrease of MEG3 activity can affect the growth of AML cells and the mechanism of AML cell growth.

TET gene plays a key role in many biological processes, including the reorganization of fertilized eggs, differentiation of pluripotent stem cells, bone marrow hematopoiesis and the development of leukemia. TET2 gene is located on chromosome 4q24, contains 11 exons, length of 150kb [25], is one of the important genes involved in surface epigenetic regulation, to methylation, plays an important role in epigenetic regulation and bone marrow in DNA. The study found in acute myeloid leukemia, myelodysplastic syndrome, chronic true bone marrow mononuclear cell leukemia, polycythemia, thrombocytosis, primary myelofibrosis, mast cell TET2 gene mutation exists in [14, 26–28]. In the AML study found that nearly 10% of patients with TET2 gene mutation, and the mutation is heterozygous mutations, wild-type allele has not changed, indicating that TET2 may play a role in inhibiting the occurrence of tumors [29]. TET2 protein can catalyze the demethylation of DNA and regulate the whole and the site. Therefore, the mutation of TET2 may lead to the imbalance of epigenetic regulation, leading to the occurrence and development of tumors. In a large scale study of primary AML, Metzeler et al. [30] found that the mutation rate of TET2 was 23%, and the mutation rate of TET2 increased with age. In our study, compared with the normal tissue expression of TET2 in AML decreased or absent, suggesting that its expression is closely related with the occurrence of AML, we observed that TET2 in AML this law in the process of change was confirmed in the experimental study of Yang [31] people, they found that TET protein expression decreased in the family liver cancer and colon cancer, breast cancer. A large number of epigenetic studies have demonstrated that TET gene mutation is involved in the pathogenesis of bone marrow malignancies [32]. The mutation was detected in the early and late stages of tumorigenesis. In addition, other studies have shown that miR-22 can affect the prognosis of breast cancer by inhibiting the expression of TET gene, and confirmed the TET gene related to tumor prognosis on the other side [32]. Our study also confirmed that miR-22 could inhibit the expression of AML and promote the increase of white blood cells by inhibiting the expression of TET2 gene. Therefore, we do not rule out the possibility that TET2 gene interacts with miRNA to achieve the function of tumor suppressor function.

Our study will analyze AML in TET2 inactivation and MEG3relationship, and further explore the relationship between TET2 and miR-22-3p/5p, the latter will continue to study MEG3, TET2, the relationship between miR-22-3p/5p, to determine the molecular mechanism of MEG3 inactivation. Our study may further reveal the molecular mechanism of AML and provide a new theoretical basis for the study of the disease.

## MATERIALS AND METHODS

### Clinical samples

With the approval of institutional review board and human ethics committee, a total of 26 AML cases between February 2014 and August 2015 in the People’s Hospital of Hainan Province which were diagnosed with AML according to the French-American-British (FAB) criteria.20 healthy volunteers in our study were randomly obtained from the Hainan. All of the tissues were histologically examined by two senior pathologists at Department of Pathology of the Hospital based on World Health Organization (WHO) criteria.

### Cell culture

The human AML cell line K562 was obtained from the Biofavor Biotech (Wuhan, China). Cells were incubated 1640 medium (GIBCO, USA), supplemented with 10% fetal bovine serum (Gibco, Auckland, New Zealand)

### DNA extraction, sodium bisulfite modification, and PCR

Genomic DNA from tissue samples was extracted using the Tissue DNA Kit (Qiagen) according to the manufacturer’s recommendations. Bisulfite modification of genomic DNA was performed using the EZ DNA Methylation Kit (Zymo Research) according to the manufacturer’s protocol. Bisulfite-treated genomic DNA was amplified in a 384-well plate using HotStarTaq Polymerase in a 5-μl reaction volume (Qiagen). PCR conditions were 94 °C for 4 min followed by 45 cycles of 95 °C for 20 s, 56 °C for 20 s, and 72 °C for 60 s, with a final extension of 72 °C for 3 min. Primer sequences are TET2FCTCCTGTTGAGTTACAACGCT;TET2 RACATGGTTGGTTCTATCCTGTTC;MEG3FTCTATGAAGACCAGTATGGCGTG; MEG3RCACTTCTTGCTGTCCTCCTG;

### Quantitative real-time PCR

Total RNA was extracted from frozen tissue samples or cells using the TRIzol reagent (Invitrogen, Shanghai, China) according to the manufacturer’s protocol. A total of 1 μg of RNA was reverse transcribed using the TIANScript RT Kit (TIANGEN, Beijing, China). Quantitative real-time PCR was performed using the BIO-RAD iQ5 Real-Time System (BIO-RAD, Hercules, CA, USA) and SYBR Green (TIANGEN) as a double-stranded DNA-specific dye. We performed the cDNA synthesis using a Thermo Script RT-qPCR System (Invitrogen). Target genes were amplified with primers designed using the Primer Premier Version 5.0 software.

### Transfection

TET2-siRNA, miRNA-22-3p mimics, miRNA-22-5p mimics, to cells for 24h for loss-of-function experiments using Lipofectamine 2000 (Invitrogen) according to the manufacturer’s instructions. The NC scrambled oligonucleotide does not encode for any known lncRNA. Transfection efficiency was verified by SYBR Green real-time PCR detection of TET2, MEG3 expression.

### Detection of cell reproductive capacity

Cells in TET2-siRNA1 group, TET2-siRNA2group, and miRNA-22-3p mimics group and miRNA-22-5p mimics, NC group, and normal group were inoculated in 96-well plate. After 24h, 36h, 48h after inoculation, 10 μl CCK8 was added per well the optical density at 450 nm was measured after 4 hour of incubation at 37.0°C using a Tecan Infinite M200 Multimode microplate reader (Mechelen, Belgium). Each experiment was performed 3 times. With 3 ventral orifices in each group, the experiment was repeated for three times.

### Statistical analysis

The SPSS 17.0 statistical software (SPSS Inc., Chicago, IL, USA) and Microsoft Excel (Microsoft Corporation, Redmond, WA, USA) were used for statistical analysis. The non-normal measurement data were represented by Median. Bilateral Mann-Whitney U test was used in comparison between two groups. They were shown as mean ± SD. To explore the correlation between TET2 and MEG3expression, two-tailed Chi-squared test was employed. Differences between the groups were compared using the Student’s t test, and results were considered statistically significant at P < 0.05.
